# The neural basis of habit formation measured in goal-directed response switching

**DOI:** 10.1016/j.isci.2025.114568

**Published:** 2026-01-02

**Authors:** Mario Michiels, Vincent Man, David Luque, Ignacio Obeso

**Affiliations:** 1 CINAC, Hospital Universitario HM Puerta del Sur, Madrid, Spain; 2 Division of the Humanities and Social Sciences, California Institute of Technology, Pasadena, CA, USA; 3 Departamento de Psicología Básica, Universidad de Málaga, Málaga, Spain; 4 Instituto de Investigación Biomédica de Málaga y Plataforma en Nanomedicina (IBIMA- Plataforma BIONAND), Málaga, Spain; 5 Cajal Neuroscience Centre, Consejo Superior de Investigaciones Científicas (CSIC), Madrid, Spain; 6 PhD Program in Neuroscience, Universidad Autónoma de Madrid-Cajal Institute, 28029 Madrid, Spain

**Keywords:** Behavioral neuroscience, Cognitive neuroscience, Medical imaging

## Abstract

Replacing a habitual action with goal-directed control involves a cost whose neural mechanisms in humans are not well established. Our study quantifies this cost and uncovers its neural correlates using fMRI and neurostimulation. Training S-R links in overtrained stimuli (compared to less trained ones, termed standard-trained stimuli) increased RT switch costs, explained by drift diffusion modeling. Training engaged sensorimotor areas and the posterior putamen, whereas standard-trained behaviors recruited the posterior caudate, insula, and prefrontal regions. A cortical network orchestrated habit expression (right S1 with the left anterior insula/prefrontal areas) while also implicating the basal ganglia when overriding habits (left premotor with the putamen). Importantly, stimulation of the left premotor cortex played a causal role in habit control, enhancing performance across both the training and devaluation phases. Our findings reveal an interaction between habitual and goal-directed brain regions, highlighting shared neural dynamics when overriding habitual behaviors.

## Highlights

• RT switch cost increases with overtraining, indexing human habit strength
• fMRI reveals cortical-striatal networks for both habit expression and overriding
• TMS shows a causal role of the premotor cortex in both habit acquisition and reversal
• Computational modeling links automaticity to drift-diffusion decision parameters

## Graphical abstract

**Figure:**
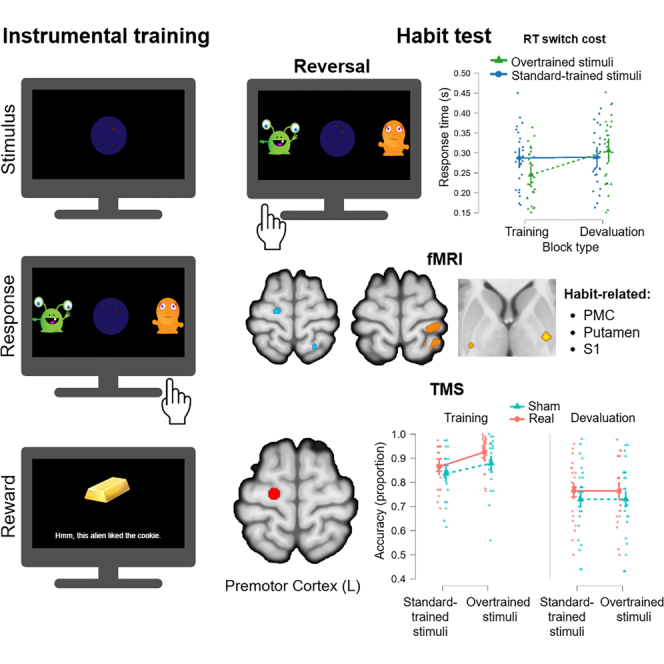

## Introduction

Changing established habits is often necessary. Successfully overriding an old habit requires considerable effort, and initially, greater mental costs arise due to the intrusion of the acquired habit. The additional cost of overriding habits reflects the conflict between two distinct, competing systems.^1^ The first is the goal-directed system, in which actions are executed by prospectively evaluating the behavioral options to achieve task goals, considering the available knowledge of the task and environmental context. Goal-directed actions are, therefore, computationally demanding and slow. The second is the habit system, where stimulus-driven responses are executed automatically. These responses are gradually acquired through repeated practice (i.e., by trial and error) and then applied in a highly efficient manner, enabling very rapid execution. However, this efficiency comes at the cost of inflexibility, making habitual behaviors difficult to override.

Recent habit research in humans has encountered difficulties in replicating key findings from animal studies, such as the relationship between habit strength and the extent of prior training.^2^^,^^3^^,^^4^ This difficulty is unsurprising, as humans are more likely to strategically adjust their behavior in response to shifting reward conditions.^5^ Nevertheless, a pioneering neuroimaging study that induced habits in humans successfully replicated animal findings using overtraining procedures.^6^ This and other studies pinpoint the posterior putamen as a critical hub to learn and retrieving habitual behaviors.^6^^,^^7^^,^^8^ Trying to decipher the cortico-subcortical connectivity, de Wit et al. (2012)^7^ reported an increased anatomical pathway from premotor cortex to posterior putamen associated with an individual’s tendency to habit-like performance. Yet, no information is available on the brain network involved in learning and overriding habitual actions. Deciphering whether similar or different neural circuits operate in expressing or reversing habits will offer a holistic view on how the habitual brain is organized (rather than isolated neural hubs) and provide insights to pathologies that implicate the habitual pathways.

Importantly, it remains unclear whether previous studies truly show the functioning of the habit system, especially when failing to demonstrate the expected overtraining effect. Recently,^9^ showed results in line with the expected overtraining effect. They manipulated the amount of training within subjects (overtrained vs. standard-trained stimuli) following a three-day training regime. Habit formation was assessed through a partial reversal test (in which one outcome was devalued) using time pressure (500 ms to respond), which could take place either on the first day (standard-trained condition) or on the third day of the experiment (overtrained condition). For the habits test, the outcome of the habitual response was devalued (outcome devaluation test^10^), so participants had to change their habitual response to obtain a still valuable outcome. The protocol reveals additional response time (RT) costs when switching habitual responses during devaluation (RT Switch cost).^9^ Importantly, this RT switch cost was higher in the overtrained condition, where habits are thought to be stronger and produce greater interference with new goal-directed responses. These findings have since been replicated by an independent pre-registered study.^11^ The increased RT cost suggests greater difficulty in modifying established habitual responses and provides a novel measure to account for habit strength in humans. Luque et al.’s^9^ test reflects the common situation in which the two decision systems compete and the more advantageous goal-directed system is finally expressed. Critically, this allows the study of the habit system without the need to artificially force habitual errors.

We hypothesized that extensive training would increase RT switch cost by engaging latent automatic components in drift-diffusion decision processes (behavioral study). At the neural level, we expected overtraining during the outcome devaluation test to recruit the cortico-striatal habit network, with dynamic interactions between cortical and subcortical regions revealed by fMRI. We further hypothesized that disrupting premotor cortex activity with TMS would alter accuracy or reaction times, thereby demonstrating a causal role of this region in habit expression (TMS study). The overall objective of this work was therefore to validate the RT-switch cost effect in overtraining, to identify its neural correlates beyond basal ganglia activation, and to establish the causal involvement of the premotor cortex in human habit control.

## Results

### Methods summary

**Figure 1:**
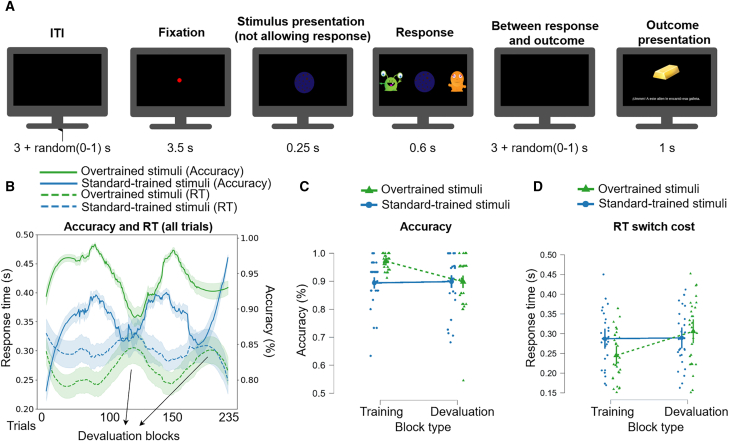
The reward learning task and behavioral results (A) Example of a single trial. On each trial, a cookie stimulus appeared at the center of the screen, and two aliens were displayed on the sides. Participants were instructed to press either the <q> (left) or <p> (right) button to choose which alien to feed. Choosing the alien whose preferences matched the presented cookie yielded an optimal outcome (gold or diamond, 100 points); the alternative response yielded a suboptimal outcome (coin, 5 points). (B) Accuracy and response time across trials, with a Savgol filter (window size = 71) applied to smooth the data for better visualization. Deviation width was reduced to 5% for clarity; shaded areas represent standard deviation. (C and D) (C) Comparison of performance between overtrained and standard-trained stimuli in terms of accuracy and (D) RT switch cost during training and devaluation blocks. Before each devaluation block, on-screen instructions specified which optimal outcome (gold or diamond) would now be worth zero points, while the other outcomes remained unchanged. During these blocks, the feedback screen displayed a question mark (“?”) instead of the earned points. Data are represented as mean ± SEM.

A reward learning task was employed (Figure 1A) with 33 participants in the fMRI experiment and 26 in the TMS experiment. Participants were trained over three days on stimulus-response links, with some stimuli receiving extensive training (overtrained) and some receiving standard training. The first two days (online sessions) consisted only of training blocks, while the last session (inside the scanner) reduced the number of training blocks and included devaluation blocks to assess habitual performance.

For each trial, one stimulus (cookie image) appeared at the center of the screen. At each side of the screen, an alien was displayed (Figure 1A). The participants’ response consisted of pressing the <p> or <q> buttons (i.e., left/right), thus earning points by feeding the aliens. For each trial, there are only two possible responses (i.e., <p> or <q>). One of them will lead to feeding the alien with his preferred cookie (optimal outcome), while the other will lead to the suboptimal outcome. The optimal outcomes were displayed as gold or diamond, both valued at 100 points. The suboptimal outcome was always displayed as a coin, valued at 5 points (Table 1). Importantly, the two alien images are only for display purposes, and they never exchange their sides. Also note that, for each cookie, it is only possible to earn one of the optimal outcomes (either gold or diamond), which remains throughout all the experiment (Table 1).

**Table 1:** S-R-O associations

Stimulus->Response->Points (outcome)	Relative frequency in training (sessions 1 and 2)	Relative frequency in devaluation (session 3, fMRI)
S1->R1->100 (gold)	×1 (standard-trained)	×1
S1->R2->5 (coin)		
S2->R2->100 (gold)	×3.5 (overtrained)	×1
S2->R1->5 (coin)		
S3->R1->100 (diamond)	×1 (standard-trained)	×1
S3->R2->5 (coin)		
S4->R2->100 (diamond)	×3.5 (overtrained)	×1
S4->R1->5 (coin)		

In each devaluation block, only one optimal outcome was devalued: the first block targeted one optimal outcome, and the second block the other.

Before each outcome devaluation block, a slide appeared on the screen, instructing participants that one of the optimal outcomes would now be zero valued. For this, the image of that outcome (either gold or diamond, counterbalanced between participants) was shown in the instructions screen. The instructions slide also explained that all the other rewards kept their value and that now the feedback screen will hide the earn points by showing a question mark (“?”) instead of the earned points. Note that there are two ways to obtain this devalued outcome (one for the overtraining stimulus, and another for the little training) (check Table 1), so this is what allows us to compare the effect of training for the devaluation phase. This way, we can also compare devalued vs. still-valued trials. Note that only one optimal outcome was devalued in each block: the first block targeted one outcome, while the second devalued the other. All the other outcomes (optimal and suboptimal) retain their original values (see Table 1).

### Habit learning dynamics

As expected, training led to improvements in establishing the correct associated answer to each cue (i.e., increased accuracy) and its speed when selecting the action (i.e., faster reaction times, RT). Consistent with optimal learning, accuracy during training was higher for overtrained stimuli compared to standard-trained stimuli (Estimate = 0.077, SE = 0.010, z = 7.853, *p* < 0.001; Figure 1B). Devalued trials revealed a reduction in accuracy for overtrained stimuli (i.e., fewer correct response switches) compared to the training phase, as indicated by a significant interaction between block × amount of training (F(1, 4850.35) = 22.981, *p* < 0.001; Figure 1C). These findings support our hypothesis that modifying habitual responses is more difficult in the overtrained condition.

Further analysis of learning dynamics revealed that response speed during training was faster for overtrained stimuli compared to standard-trained stimuli (Estimate = −0.045, SE = 0.004, z = −10.103, *p* < 0.001; Figure 1D). On devalued trials, participants showed longer RTs for response switches in the overtrained condition compared to the training phase (interaction block × amount of training: (F(1, 27.04) = 20.936, *p* < 0.001; Figure 1D). These findings suggest that modifying a habitual response incurs greater cognitive costs in terms of RT for overtrained stimuli. These results are consistent with our hypothesis and previous findings demonstrating that RT Switch costs are greater for extensively trained stimuli.

### Drift diffusion processes in habit expression and modulation

The final session of the fMRI experiment was used to fit the HDDM to both training and devaluation blocks. Following the model fitting, a posterior predictive check confirmed that the complete model (i.e., including all variables) successfully replicated the behavioral data across all conditions (training and devaluation blocks for both standard and overtrained stimuli; Figure S1). To assess model performance, we fitted five alternative model variations, each with a reduced set of parameters (separating conditions for *t*, *a*, *v*, and *t* = 0). Model comparison based on the DIC indicated that the complete model (*DIC* = −9054.27), which accounted for training and devaluation blocks as well as overtrained and standard-trained stimuli across all parameters (*t*, *v*, *a*), provided the best fit (Table S7). However, parameter recovery analysis revealed non-singular parameters, indicating that multiple parameter combinations could produce equivalent model fits in terms of global likelihood. While this limits the direct interpretation of individual parameters, the successful fit to a drift diffusion model suggests that our study captured systematic changes in decision-making processes, consistent with an accumulation-to-threshold mechanism typical of automated behaviors.^12^

### The role of the sensorimotor circuit in habit expression and goal-directed response switching

**Figure 2:**
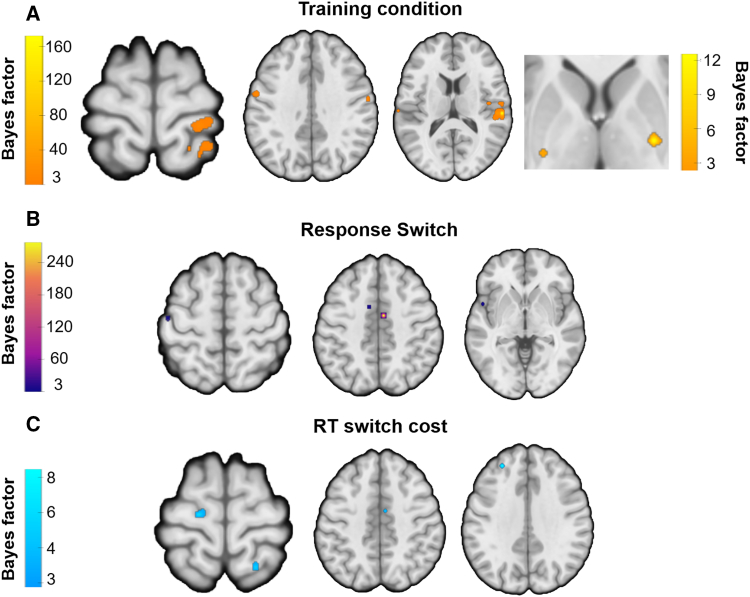
Brain activity during training and devaluation in habitual behavior (overtrained vs. standard training) A Bayes factor ≥ 3 was used for thresholding. (A) GLM univariate contrasts show the activation of habitual brain areas during training. Striatal activation in the rightmost axial view was identified using a small volume corrected analysis with a striatal ROI (color scale shown on the right). (B and C) (B) GLM univariate contrasts highlight the activation of habitual brain areas when switching the overtrained S-R associations, and (C) incorporating RT switch costs as a regressor to model BOLD data. For the complete pattern of results, see Tables S1–S3. No cluster extent threshold was applied, except for the first contrast, where single-voxel clusters were removed for visualization purposes due to the higher number of trials.

**Figure 3:**
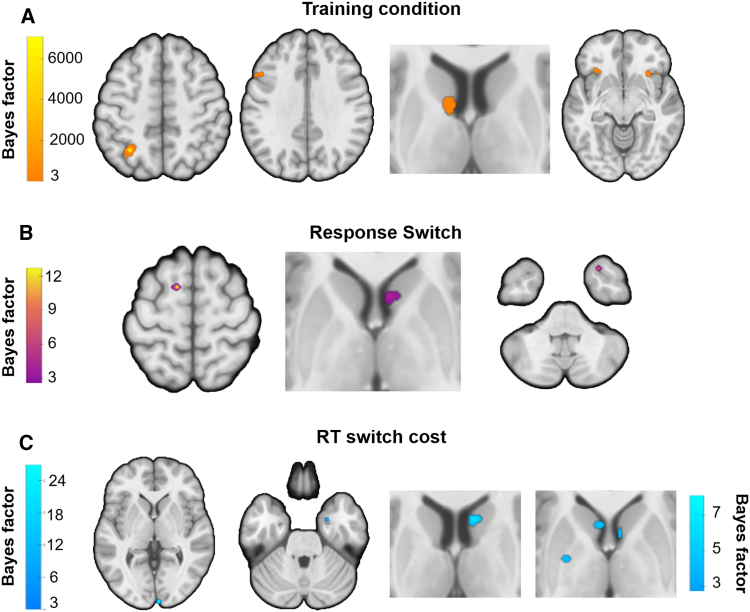
Brain activity during training and devaluation in goal-directed behavior (standard training vs. overtrained) A Bayes factor ≥ 3 was used for thresholding (as seen in the color bars). (A–C) (A) GLM univariate contrasts show goal-directed activation during training; (B) GLM univariate contrasts engaging goal-directed brain areas when switching the overtrained S-R associations, and (C) when adding RT switch costs to model the BOLD data as regressor; the striatal activation in the right side used a small volume corrected analysis with a striatal ROI (color scale shown below the main one). Other additional regions are displayed in Tables S1–S3B. No cluster extent threshold was applied, except for the first contrast, where we had more trials, so we removed single-voxel clusters for visual purposes.

Bayesian statistics with multiple comparison correction were used in both univariate and g-PPI connectivity analyses. Results reveal that in the training phase, overtrained stimuli (compared to standard-trained stimuli) elicited activity in the primary and secondary somatosensory cortex (bilateral), right superior temporal gyrus, and left primary motor area (Figure 2A; Table S1A). Whole-brain analysis did not reveal habit-related striatal activation. However, given the *a priori* significance of this area and following previous research (see Guida et al., 2022), we implemented a small volume correction (SVC) analysis using a striatum region of interest (ROI), which revealed bilateral activation in the posterior putamen (Figure 2A; Table S1A). To further investigate neural activity associated with goal-directed processes during training, we analyzed the inverse contrast (standard > overtrained stimuli). This analysis revealed activation in the bilateral posterior caudate, bilateral insula, left inferior parietal lobule, left dorsolateral prefrontal cortex (dlPFC), left orbitofrontal cortex (OFC), and left fusiform area (Figure 3A; Table S1B).

During the devaluation phase, neural activity related to the intrusion of well-learned habits (response switch overtrained > standard-trained) revealed activation of the left secondary motor cortex, left superior parietal lobule, premotor cortex, and motor cortex (Figure 2B; Table S2A), suggesting habitual activity from the sensorimotor circuit. For the inverse contrast (response switch standard-trained > overtrained), we observed activation in the bilateral caudate, right vlPFC, and right hippocampus/amygdala (Figure 3B; Table S2B). To further examine the neural correlates of RT switch costs as habits wore off, we regressed the RT costs (RT switch cost overtrained > standard-trained), which revealed activation in the left PMC, right superior parietal, left dlPFC, and mid-cingulate (Figure 2C; Table S3A). No striatal activation was observed in either whole-brain or ROI analyses. The inverse contrast (RT switch cost: standard-trained > overtrained) showed activation in right V1, left dlPFC, right ventrolateral prefrontal cortex (vlPFC), left inferior temporal cortex, right fusiform area, and right hippocampus/amygdala (Figure 3C; Table S3B). ROI analysis of the striatum additionally revealed activation in the bilateral caudate, left pallidum, and right anterior putamen (Figure 3C; Table S3B). At feedback, expected findings were obtained during reward receipts, including the anterior cingulate and amygdala for overtrained stimuli (Table S8).

### Habit hubs activity explains the degree of switch costs

Individual differences in habit expression and modulation have been shown along cortico-subcortical hubs. Individuals more prone toward goal-directed behavior largely depend on fronto-parietal regions (unlike those showing greater reliance on habitual behaviors),^2^ while stronger tracts between left premotor and posterior putamen predicted greater habit perseverance.^7^ As individuals may engage in different strategic forms, we hypothesized that brain activity in areas engaged with habitual responses (during S-R training) would be associated with the individual magnitude of RT Switch costs (at devaluation).

To test this, we correlated specific brain activity from the habitual contrast in the training phase (overtrained > standard-trained) with RT switch cost (baselined with respect to training performance). This analysis was conducted across all regions identified in the univariate contrasts to explore how training-related performance may impact subsequent habitual behavior in the devaluation phase. No significant correlations survived multiple comparison corrections (Bonferroni, false discovery rate), likely due to the stringent corrected significance threshold required for testing multiple ROIs (>25). Consequently, some significant correlations may not have been detected. Therefore, we also report findings based on uncorrected *p*-values.

**Figure 4:**
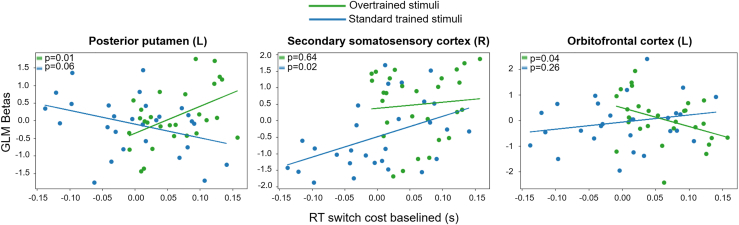
Brain activity during training correlates with switching during devaluation GLM beta values during training (overtrained > standard-trained) correlated with RT switch cost (uncorrected). The plots only show the brain regions with significant correlations with RT switch costs, displaying the *p*-values for both overtrained and standard-trained conditions.

We observed that participants with greater activation in the left posterior putamen exhibited more habitual behavior in the devaluation phase (i.e., larger RT switch cost) (r = 0.48, *p* = 0.01; Figure 4), suggesting that higher left putaminal activation may be associated with greater intrusion of habits. Interestingly, the opposite pattern was observed for standard-trained stimuli (Figure 4; r = −0.36; *p* = 0.06). A similar pattern was found in the right secondary somatosensory cortex, but only for standard-trained stimuli (r = 0.44; *p* = 0.02; Figure 4). This may suggest that higher somatosensory cortex activity during training facilitates the better encoding of less trained (generally weaker) associations, leading to a higher switching cost during devaluation. Conversely, in the left OFC, participants with higher activation during training for overtrained stimuli demonstrated greater flexibility and less habitual behavior during devaluation (r = −0.38; *p* = 0.04; Figure 4), suggesting a stronger engagement of goal-directed processes in the OFC.

For completeness, we also examined the correlations between training-phase betas and raw RTs during devaluation (i.e., without subtracting training RTs). These associations followed the same direction as the RT switch cost effects but did not reach significance in any ROI (posterior putamen: overtrained r = 0.32, *p* = 0.1; standard-trained r = −0.3, *p* = 0.12; S2: overtrained r = −0.03, *p* = 0.87689; standard-trained r = 0.13, *p* = 0.5; OFC: overtrained r = −0.13, *p* = 0.5; standard-trained r = 0.06, *p* = 0.75). These weaker effects likely reflect the additional variability present in raw RTs, which we hypothesize could be unrelated to habitual interference (e.g., familiarity with the stimuli, boredom), especially given that the validity of using the baselined measure was already confirmed in all our other behavioral analyses.

### Cortico-subcortical interactions when expressing and modulating habits

**Figure 5:**
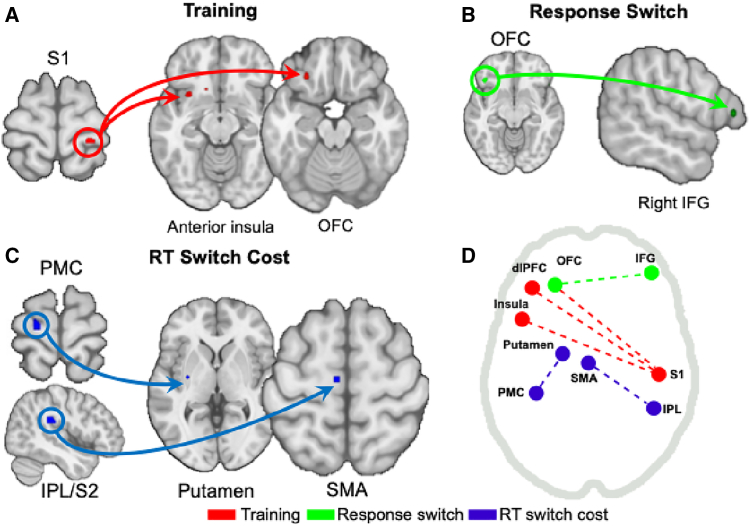
Functional connectivity findings in habit expression and modulation (A–C) Brain-wide connectivity patterns associated with (A) training, (B) response switching, and (C) RT-switch cost conditions. The diagram illustrates interactions between regions identified in the univariate contrasts (training, switching, and RT Switch cost) as revealed by the g-PPI analysis. (D) Summary of the main connectivity findings across conditions for each specific contrast with significant connectivity.

To date, no clear habitual circuitry is available in the literature, nor is it clear how transitions to new networks occur when overriding habits is required. To investigate how functional connectivity shifts during habitual behaviors and their modulation (i.e., goal-directed switching), we selected different seed regions for overtrained against standard-trained trials (using g-PPI): the right primary somatosensory cortex (S1) for the training condition, the motor cortex and left orbitofrontal cortex (OFC) for the switching condition, and the left premotor cortex (PMC) and right secondary somatosensory/inferior parietal lobule (S2/IPL) for the RT switch cost regressor. During training, using the right S1 as the seed region revealed significant connectivity with the left anterior insula, left OFC, and left DLPFC (Figure 5A; Table S4A). When switching overtrained responses (compared to switching standard-trained responses), the motor cortex seed showed a functional connection with the right hippocampus/amygdala (Table S4B). This contrast also revealed connectivity between left OFC (seed) and right vlPFC (Figure 5B; Table S5). In the RT switch cost analysis, we observed significant connectivity between the left PMC and left putamen, as well as between the right S2/IPL and left SMA (Figure 5C; Table S6). These interactions are summarized in Figure 5D for clarity.

### Causal contribution of the premotor cortex to habits

**Figure 6:**
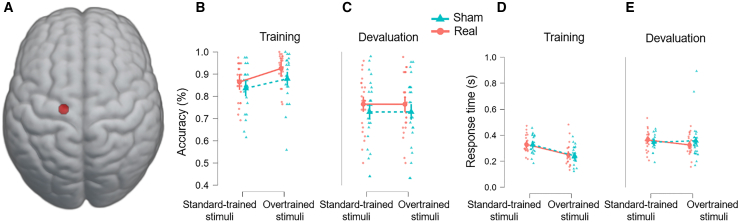
Behavioral findings and neuromodulation (real vs. sham TMS) for overtrained and standard-trained stimuli (A–C) (A) TMS stimulation target identified from GLM analysis, highlighting the left PMC; (B) behavioral results following TMS, showing accuracy in real vs. sham conditions in the training; and (C) devaluation conditions. (D and E) (D) RT in training and (E) during the devaluation phase for both stimulus types. Data are represented as mean ± SEM.

To establish a direct link between our imaging findings and the behavioral activation of habits, we selectively modulated the left PMC (Figure 6A) to induce changes in either habit expression (training) or modulation (devaluation). The PMC is a key cortical area engaged in habitual control connected to the putamen^7^ and a putative target to modulate the expression of habits. The overall results (i.e., without distinguishing between real and sham conditions) replicated the overtraining effects observed in the fMRI experiment in terms of accuracy and RT Switch cost (see supplemental information). Importantly, we observed a global improvement in accuracy when real TMS was applied (compared to sham) as indicated by a significant main effect of TMS (*F*(1, 11,718) = 25.781, *p* < 0.001; Figures 6B and 5C). This enhancement was present across task conditions, suggesting that training was performed at a higher level following real TMS compared to sham stimulation (Figure 6B), an effect that also extended to devaluation tests (Figure 6C). This dual effect has important implications for how the brain executes habits, indicating that the left PMC contributes not only to habit expression but also when habits are successfully overridden by goal-directed control. In other words, the left PMC plays a biological role in both the implementation of habitual responses and their modulation by competing goal-directed processes. However, this improved accuracy did not result in significant differences in reaction times during either training (Figure 6D) or devaluation (Figure 6E). To control for potential motor side effects of stimulation, a finger-tapping task was conducted, revealing a significant main effect of hand (F(1, 1261) = 216.237, *p* < 0.001; faster right-hand responses), but no significant interaction between TMS and hand dominance.

## Discussion

Recent studies have failed to consistently replicate the expected relationship between the amount of training and habit strength, limiting translational research between human and animal models. In this study, we addressed these issues by assessing habit strength based on its interference with ongoing, incompatible goal-directed actions, specifically when participants were required to change their habitual responses under time pressure. The effectiveness of our paradigm stems from the focus on measuring response conflicts rather than direct choices (or the absence of choice), as is typically done in conventional devaluation methods.^7^ The switch cost measure (with response-time constraints) effectively captured increased habit strength by revealing delayed response times when participants were required to break established S-R associations.^9^ Additionally, the HDDM model decomposition of our behavioral data aligns with the notion that training leads to rapid response selection.^13^^,^^14^ These findings suggest that drifts and evidence accumulation processes become automatized during habit expression and modulation (Ratcliff & Frank, 2012). Our results confirm that training, coupled with time pressure, plays a critical role in limiting goal-directed control, enabling a clearer manifestation of habitual tendencies.^15^

Importantly, we argue that during the devaluation phase, it is not required for overtrained performance to fall below the standard-trained condition. Rather, a decrease in performance within the overtrained condition itself is sufficient to demonstrate a habit-related cost. The benefit gained through extended practice is lost once contingencies are altered, revealing reduced flexibility. Statistically, this pattern is best captured by the interaction between the amount of training and block type. This effect is directly tested and maximized by our within-subjects design and linear mixed-effects model. Without considering this interaction, the cost would remain hidden. Since only the overtrained condition developed such a performance advantage in training blocks, it is therefore the only condition showing a selective decline. This interpretation aligns with prior human studies, where habit effects are characterized by the loss of a training-related benefit rather than performance dropping below standard-trained levels.^2^^,^^15^^,^^16^^,^^17^ This pattern differs from the classical criterion used in non-human animal studies, where habits are inferred only when overtrained performance becomes worse than performance after more limited training once contingencies or reinforcer value are changed.

Additional cognitive layers may be at stake. Executive control processes are needed when S-R mappings need to be reversed.^18^ Yet, during devaluation tests, the S-R rule stays the same, and only outcome value changes, being the overtrained response still automatically triggered. This automatic S-R retrieval shall compete with executive control and goal-directed new response, a metric that was embedded within our switch cost RT results. This slowdown, therefore, reflects habit intrusion with a probable impact of executive control that was not directly tested here.

The mechanisms underlying how the human brain navigates habits and shifts from goal-directed to habitual behavior remain unclear. Building on current behavioral theories concerning the transition from habitual to goal-directed control, our findings suggest the potential joint processing of habitual and goal-directed cues. In line with several studies, we observed activation in the sensorimotor cortex and putamen during training.^2^^,^^6^^,^^7^^,^^19^ However, this pattern is not always reported in the literature.^20^^,^^21^ We propose that the robust evidence for the role of the putamen in activating habitual behavior in our study stems from the strong habitual component of actions, which was promoted by both the training regime and the imposed response time constraints. The engagement of the cortico-subcortical neural system was further reflected in the strong effect of overtrained stimuli during training, which importantly induced longer switching costs (demonstrated by positive correlations with both putamen and S1). This result confirms a critical link between sensorimotor control during training and its subsequent impact when habits must be overridden, highlighting a dynamic interaction between habitual and goal-directed processes.

When behavior operates in habitual mode, control-related brain areas likely monitor signals of updated contextual information to determine whether to maintain or override ongoing habits. The best-fitting DDM model showed differences in diffusion and threshold variables in a similar manner for both training and devaluation blocks, further supporting this notion. Moreover, the primary somatosensory cortex (S1) contributes to habit organization by interacting with regions typically associated with goal-directed control, such as the anterior-insular cortex^22^ and visual cortex.^23^ Similarly, the observed connectivity between the secondary somatosensory cortex (S2)/inferior parietal lobe (IPL) and the SMA, as well as between the M1 and the hippocampus/amygdala, underscores the complex interplay between motor execution, planning, and memory processing when behavior is governed by habitual control. These findings suggest that the somatosensory cortex is part of a broader network involved in integrating sensory inputs and emotional salience with ongoing motor responses. This network is crucial for both habitual and goal-directed control, facilitating the coordination of actions based on past experiences and current goals.^24^^,^^25^

A long-standing question in the literature is how the brain overrides habitual behaviors. Identifying the neural mechanisms that disengage habits when they are no longer needed is critical for understanding the flexibility of the habitual system and when this becomes dysfunctional (as in pathological conditions such as addiction or OCD). Our findings provide evidence for a predominantly motor-related cortical network involved in both the expression and modification of overtrained habits, including the left PMC, S2/IPL, left SMA, IFG, and cingulate cortices. In particular, connectivity analyses highlighted the interaction between the PMC and putamen, supporting previous findings linking putaminal activity to habitual behavior.^23^ However, given that habits are no longer adaptive during devaluation (i.e., when they begin to wear off), both goal-directed and habitual control mechanisms require co-existence in the brain to successfully guide adaptive behavior. We have identified a key transition from habitual behavior mediated by the right IFG, previously implicated in behavioral control,^26^ and the middle cingulate cortex, which is involved in motor planning and adaptive control.^27^ The IFG, in particular, is strongly associated with goal-directed processes, suggesting its involvement in both habitual and goal-directed actions (or the transition between the two). These regions appear to be essential for overriding established habitual responses and represent potential targets for neuromodulation protocols in disorders characterized by the overexpression of habits.

Regarding the cingulate cortex, a large body of literature has linked this region with habitual behavior, with studies implicating different sections along the anterior-to-posterior axis depending on the specific function examined.^21^^,^^28^^,^^29^^,^^30^ In our study, the posterior cingulate was engaged during training in the overtrained condition, whereas habit switching elicited a more anterior activation pattern.^21^ This suggests that cingulate involvement in habit expression and modulation may occur at distinct anatomical locations, depending on the behavioral context. Critically, no striatal activation was observed in the overtrained conditions during devaluation, either in whole-brain or ROI analyses. One intriguing possibility is that extended training facilitated a transfer of control from the basal ganglia to cortico-cortical projections, consistent with the neurobiological model of automaticity proposed by Ashby et al. (2007).^31^ Under this framework, as habits become ingrained, their expression may rely more on cortical connectivity, particularly in novel situations where habitual responses must be overridden. However, despite this shift, residual subcortical activity may continue to exert influence, leading to intrusions from previously reinforced habit-related regions.

To investigate the cortical role in habit expression and modulation, we applied cTBS (inhibitory) over the left PMC (comparing its effects to a sham condition). Overall, cTBS led to an improvement in accuracy across both training and devaluation blocks (yet pure motor movement remains intact; see finger-tapping task results above). This improvement was likely driven by the inhibition of habitual circuitry, prompting participants to adopt a more goal-directed and controlled response strategy, even during training, a phase typically observed to foster habitual control. Indeed, previous attempts in the selective suppression of habitual circuit areas (e.g., infralimbic cortex in rats) resulted in enhanced goal-directed responding.^32^ Selective inhibition of infralimbic activity immediately after each lever press, at a time when animals are relying on a habitual strategy, restored sensitivity to contingency degradation and effectively made behavior run under goal-directed control.^32^ Hence, while other area was stimulated and other species/protocols were tested, it may all indicate that blocking the habit system actively recruits goal-directed control. At the physiological level and given the current data, it is important to acknowledge that no single interpretation can be firmly drawn, and most explanations remain nonspecific (e.g., changes in neural excitability, motivation, attention, and so on). Without an interaction or other differential effect across conditions, interpretation is necessarily limited. The following considerations are therefore speculative and intended only to provide the reader with plausible frameworks.

We argue that the presumed reduction in PMC excitability might have increased the influence of goal-directed decision-making processes, thereby improving accuracy. This interpretation is consistent with the role of the posterior putamen-PMC pathway in habit formation^32^ and with previous accounts suggesting how PMC stimulation might modulate putaminal activity,^33^ effectively suppressing habit-related signals associated with well-learned cues. Compatible with the above interpretation, the local effects of PMC stimulation may have enhanced participants' overall cognitive control or attentional mechanisms (similar to.^34^ This interpretation aligns well with research suggesting that the PMC is involved in cognitive control processes, including the selection and initiation of actions based on current goals.^34^

In contrast, an alternative explanation is that PMC stimulation enhances putaminal functions, which seems less probable, as habitual behaviors typically plateau with consistent training, limiting further improvements in performance. Overall, both options are plausible, given that a reduction in putamen activity during habitual behavior may have disrupted the balance between habitual and goal-directed processes, potentially increasing cognitive control demands. Altogether, the PMC seems to be involved in both the expression and overriding of habits, thereby suggesting its broader role when the management of habit cues is critical to adapt behavior.

### Limitations of the study

We note that the sample was largely composed of young female psychology students. This demographic composition does not compromise the internal interpretation of the results, given the strict exclusion of individuals with psychological or neurological conditions; however, it may limit the extent to which the findings can be generalized to older groups or populations with different demographic or clinical profiles.

Regarding the TMS protocol, we targeted a single cortical site (left PMC), which constrains causal inference about the broader cortico-subcortical network implicated in habit expression and override. Finally, in some cases, the MRI session occurred before the TMS session, raising a minor technical concern because it is unclear whether the MRI’s static field might alter TMS influence. However, this uncertainty was partly mitigated by the counterbalanced order of sessions across participants.

## Resource availability

### Lead contact

Further information and requests for data access should be directed to the lead contact, Mario Michiels (mario.michiels@estudiante.uam.es).

### Materials availability

This article did not generate new unique materials apart from the equations described in the behavioral analysis section.

### Data and code availability

• Data: Behavioral, TMS data, and analysis scripts are available at https://osf.io/w86nt/files/osfstorage. fMRI data are available upon reasonable request.
• Code: Analysis scripts are provided in the OSF repository.
• Additional information: Any additional information needed to reanalyze the data is available from the lead contact upon request.

## Acknowledgments

We are grateful to Pasqualina Guida and David Mata-Marín (CINAC, Hospital Universitario HM Puerta del Sur, Móstoles) for their help in the fMRI data acquisition. MM was funded by 10.13039/501100004587the Instituto de Salud Carlos III (PFIS contract) and Fundación HM Hospitales. IO was funded by the 10.13039/501100004587Instituto de Salud Carlos III (Miguel Servet, CP18/00038). This research has been funded by 10.13039/501100004587ISCIII (PI19/00298) from the 10.13039/501100004837Ministry of Science and Innovation and PROYEXCEL_00287, awarded by the 10.13039/501100011011Junta de Andalucía (Spain). The funders played no role in the idea, design, data collection, analysis, decision to publish, or article editing and writing.

## Author contributions

M.M. design, data collection, analysis, writing; V.M: DDM analysis, writing; D.L. design, data collection, writing; I.O. design, data collection, writing.

## Declaration of interests

The authors declare no competing interests.

## Declaration of generative AI and AI-assisted technologies in the writing process

AI-assisted tools (ChatGPT 5, OpenAI) were used solely to improve grammar and phrasing of some sentences. All content, analyses, and scientific interpretations were created and verified by the authors, who take full responsibility for the article.

## STAR★Methods

### Key resources table

**Table:** 

REAGENT or RESOURCE	SOURCE	IDENTIFIER
**Deposited data**		

Anonymized behavioral and TMS data	This paper	https://osf.io/w86nt/files/osfstorage
fMRI data	This paper	Upon request

**Software and algorithms**		

Python	Python 3.8	https://www.python.org/
JASP	JASP 0.19.1	https://jasp-stats.org/
HDDM	HDDM 0.9.8	https://hddm.readthedocs.io/en/latest/
nilearn	Nilearn 0.6.2	https://nilearn.github.io/stable/index.html
fMRIprep	fMRIPrep 20.2.1	https://fmriprep.org/en/stable/
RT Switch cost	This paper	NA

### Experimental model and study participant details

Experiment 1 (fMRI study) initially recruited 33 participants from the Universidad Autónoma de Madrid (mean age = 20.6 years, SD = 3.2; 29 right-handed; 22 female). As an inclusion criterion, participants were required to have no history of neurological disorders, as confirmed through self-reported questionnaires. Individuals with mild-to-severe neurological conditions were excluded from the study, including those with moderate disorders requiring medication. Four participants were excluded from the final analysis: three due to excessive head movement during the fMRI session (see fMRI analysis section for details) and one due to low performance in the devaluation block. The final analyzed sample consisted of 28 participants.

For Experiment 2, 26 participants were recruited from the Universidad Complutense de Madrid (mean age = 23.0 years, SD 4.5; all right-handed; 20 female) to complete two TMS sessions (real and sham). To ensure that participants fully understood the devaluation procedure, each block included three validation trials in which they freely chose between the rewards. Given that Experiment 2 involved four devaluation blocks (due to the inclusion of both real and sham TMS sessions), compared to only two in Experiment 1, we adjusted the exclusion criteria. Participants were excluded only if they committed ≥1 consumption trial error in ≥2 devaluation blocks (instead of ≥1 error in a single block, as in Experiment 1). Based on this criterion, one participant was excluded, resulting in a final sample of 25 participants.

The study protocol was reviewed and approved by the *Comité Ético de Investigación con medicamentos* (CEIm) of HM Hospitales (Approval code: CEIm HM Hospitales 22.11.1190E4-GHM; Approval date: 23 November 2022; Meeting Act No. 262; Protocol version 3.0, dated 5 November 2022). All participants provided written informed consent in accordance with the Declaration of Helsinki. Signed consent forms are securely stored in accordance with institutional and legal requirements.

### Method details

#### Reward learning task

An instrumental learning task adapted from^35^ was used across experiments. Each trial began with a fixation cross (500 ms), followed by the presentation of a cookie stimulus at the center of the screen. Two alien characters were displayed on either side (Figure 1), and participants indicated their choice by pressing the <q> key (left alien) or the <p> key (right alien) (Figure 1A). Each alien was consistently associated with one type of cookie; selecting the correct option produced a favorable outcome (gold or diamond; 100 points), whereas an incorrect choice produced an unfavorable outcome (coin; 5 points) (Table 1). Participants were required to respond within 600 ms; late responses triggered a timeout screen, invalidating the trial. This time limit was included to bias performance toward habitual control by constraining deliberative processes.^36^

Four cookie stimuli were used, distinguishable only by their color (brown, blue, purple, and yellow). The alien images served only as a cover story to support memory for the associations, ensuring that choices were not perceived as arbitrary left–right responses. Alien positions were fixed across the experiment and never switched sides. Each cookie stimulus was consistently paired with only one optimal outcome (either gold or diamond), which remained unchanged across all sessions (Table 1).

During outcome devaluation blocks, one of the two high-value outcomes (gold or diamond) was devalued to zero points. Before each block, participants were explicitly instructed which outcome was devalued, while being reminded that the other outcomes retained their value. In these blocks, the feedback screen showed a question mark (“?”) instead of the numerical points. Because the devalued outcome could be reached via both an overtrained stimulus and a standard-trained stimulus, the design enabled a direct comparison of the effect of training history during devaluation, as well as a contrast between devalued and still-valued trials.

To encourage motivation and attention, participants were informed that the six highest-scoring individuals would receive a real-life monetary bonus of 25 €. In Experiment 2 (TMS), participants followed the same devaluation block structure in two separate sessions (real vs. sham stimulation), held at least one week apart.

#### Experimental design

The reward learning task was employed with specific adaptations to meet the requirements of each study while maintaining identical outcome measures. Experiment 1 consisted of three consecutive sessions conducted over three days. The first two sessions were completed online, while the final session took place entirely inside the scanner, including both training and devaluation conditions. After the first session, participants’ accuracy was assessed, and those scoring ≤70% were excluded from further participation. Each online session included four training blocks, with a total of 44 trials per block: 34 trials featuring overtrained stimuli and 5 trials featuring standard-trained stimuli, amounting to 396 trials in total. Additionally, a devaluation block of four trials was introduced between the third and fourth training blocks. The inclusion of the devaluation block served to ensure that participants paid attention to the stimulus-response (S-R) associations that yielded the best rewards. This design reinforced the need to recall specific S-R pairings rather than relying solely on simple left-right decision-making strategies.

The final session was conducted inside the scanner. Participants first completed two training blocks with the same configurations as in the previous sessions. Next, a devaluation block with 44 trials (11 trials per condition: overtrained and standard-trained stimuli) was introduced, in which one of the trained associations (overtrained or standard-trained) was devalued (counterbalanced across participants). To avoid confusion between devaluation effects and regular outcomes, another training block followed, using the same configurations as the previous training blocks. Finally, a second devaluation block was introduced, devaluing the other condition (overtrained or standard-trained), while the previously devalued stimulus maintained its original value.

Experiment 2 consisted of two neuromodulation sessions (real and sham TMS) conducted in the laboratory using a within-subject design. Before each laboratory TMS session, participants completed two online sessions, followed by a final session in the laboratory. These sessions were conducted on consecutive days for each TMS condition. The task conditions remained identical to those described in the previous section.

### Quantification and statistical analysis

#### Behavioral analysis

A linear mixed-effects model (LMM) was used to test the statistical significance of the behavioral effects across both experiments.

For the response time (RT) analysis, we implemented a maximal random effects structure justified by our experimental design, as follows:

response_time∼block_type∗overtraining_stim+(1+block_type∗overtraining_stim|participant)

From now on, we will refer to this model as the RT switch cost paradigm.

For the response accuracy analysis, the model was structured as follows:

response_accuracy∼block_type∗overtraining_stim+(1|participant)

In Experiment 2, RT was analyzed as follows:

response_time∼block_type∗is_overtraining_stim∗condition+(1|participant).

The model for accuracy was defined as:

response_accuracy∼block_type∗is_overtraining_stim∗condition+(1|participant)

#### fMRI univariate contrasts

Univariate contrasts were computed using nilearn 0.4.2^37^ from stimulus onset (zero duration). Multiple univariate contrasts were conducted to account for the different task conditions.

a) **Training phase**: During the training phase, we were interested in observing the learning differences between the overtrained and standard-trained stimuli. To achieve this, we compared responses when overtrained stimuli were presented versus when standard-trained stimuli were shown (only considering the correct responses). This contrast is referred to as “*Response overtrained correct > Response not overtrained correct.*” We assume this contrast primarily reflects habitual processes, while goal-directed processes may emerge when the contrast is inverted.
b) **Devaluation phase**: The following contrasts were designed to assess the RT switch cost hypothesis, which has not been previously studied using fMRI. The initial contrast derived from this condition is *“Response switch overtrained > Response switch not overtrained.”* Note, however, that this contrast is not the most precise for testing the RT switch cost hypothesis since it includes all response switch trials, even those that did not show an additional RT cost. Nonetheless, due to the limited number of trials, this contrast provides a more reliable method for testing the hypothesis.

We also analyzed a more refined version of this hypothesis: *“RT switch cost baselined positive overtrained > RT switch cost baselined positive not overtrained.”* This contrast, a subset of the previous one, incorporates an RT baseline from the final training block (i.e., RTs from the last training block were subtracted from RTs in the devaluation blocks). Although this contrast includes fewer trials, applying Bayesian analysis allowed for reliable results that complemented the broader contrast. We assume both contrasts primarily reveal habitual activation, while goal-directed processes are expected when the contrast is inverted.

Bayesian statistics with multiple comparison correction was employed using the BayesFactorFMRI package in Python^38^^,^^39^^,^^40^ for both univariate and g-ppi analyses. The primary advantage of using Bayesian statistics is the enhanced sensitivity for studies with limited trials, such as our devaluation blocks.^41^ This enhanced sensitivity is particularly important for our study since it is well known that fMRI contrasts between purely cognitive conditions with the same sensorial inputs and motor outputs typically yield weaker activations.^42^ Moreover, in the devaluation blocks we intentionally balanced the number of overlearned and standard-trained trials to enhance discriminability (Table 1). Full technical details of the fMRI univariate analyses can be found in the supplemental information.

#### fMRI connectivity analysis: G-PPI

To examine cortico-subcortical interactions during habit expression, we conducted functional connectivity analysis using a generalized psycho-physiological interactions model (g-PPI).^43^ This analysis aimed to provide additional hints concerning whether a particular region is involved in habitual or goal-directed circuitry or whether there is an interaction between the two. Hence, the contrasts used for this analysis were the same as those described in the univariate contrasts section above. A detailed explanation of how the regressors were included can be found in the supplemental information.

#### TMS protocol

In Experiment 2, we aimed to target a key hub of the habitual circuitry using continuous theta-burst stimulation (cTBS) over the left PMC (technical details can be found in the supplemental information). The experimental procedure was divided into two phases. In the first phase, participants completed a training session at home over two consecutive days. On the third day, TMS was administered in either active or sham conditions (using a within-subject counterbalanced, single-blind design). Immediately after cTBS, participants engaged in a 5-min finger-tapping task, which served as a control condition to ensure minimal interference with M1. Subsequently, they completed the reward learning task, including training and devaluation conditions. An interval of two to three weeks was introduced between the two phases to reduce the retention of associations formed during the first phase. This interval is longer than that outlined in the standard safety guidelines for TMS (i.e., a one-week interval between sessions).^43^

During the second phase (real or sham, depending on the counterbalanced order), participants were exposed to novel stimuli characterized by different shapes and colors to ensure the formation of new associations. The TMS condition assigned to each participant in the second phase was the inverse of what they had received in the first phase, ensuring a counterbalanced design.
